# A combination of postmortem ageing and sous vide cooking following by blowtorching and oven roasting for improving the eating quality and acceptance of low quality grade Hanwoo striploin

**DOI:** 10.5713/ajas.19.0667

**Published:** 2019-11-12

**Authors:** Seung-Hoon Jwa, Yong-An Kim, Van-Ba Hoa, In-Ho Hwang

**Affiliations:** 1 Department of Animal Science, Chonbuk National University, Jeonju 54896, Korea; 2 National Institute of Animal Science, Rural Development Administration, Wanju 55365, Korea

**Keywords:** Striploin, Sous Vide, Roasting, Blowtorching, Tenderness, Flavor

## Abstract

**Objective:**

It is well recognized that beef cuts from a low quality grade are usually associated with tougher, drier and less flavorful. Thus, the present study aimed at investigating the combined effects of postmortem ageing and sous vide (SV) cooking followed by oven roasting or blowtorching on the eating quality of low quality grade Hanwoo beef striploins.

**Methods:**

Hanwoo beef striploins (quality grade 3) obtained from 36 month-old Hanwoo steers were used, and the samples were chiller aged for 0 and 14 d at 4°C. After ageing, the samples were prepared into 2.5-cm steaks which were then SV cooked at 55°C for 5 h and then raised to 60°C for 1 h, and thereafter the SV-cooked the steaks were further roasted in oven for 20 min (SV+OV) or blowtorched (SV+TC) for 2 min. The cooked samples were analyzed for microbiological quality, browning index, Wanrner-Bratzler shear force (WBSF), aroma flavor compounds and sensory properties.

**Results:**

The SV cooking significantly reduced the WBSF values in beef samples (p<0.05). Blowtorching after SV cooking led to a browner surface of the beef steaks (p<0.05). The samples treated with SV+OV or SV+TC exhibited higher levels of Maillard reaction-derived aroma flavor compounds such as; pyrazines and sulfur-containing compounds compared to those just SV cooked. More especially, the SV+OV− or SV+TC− treated samples presented significantly higher flavor and overall acceptability scores compared to those just SV cooked (p<0.05). Ageing beef for 14 d significantly improved the tenderness by reducing the WBSF and increasing the tenderness scores.

**Conclusion:**

Thus, the combination of postmortem ageing and SV cooking followed by additional treatments (blowtorching or oven roasting) could be used to improve the eating quality especially tenderness and flavor as well as overall acceptability of low grade Hanwoo beef.

## INTRODUCTION

Hanwoo beef is one of the most popular cattle meat types in Korea due to its unique palatability [1,2]. The most important factor making Hanwoo beef more palatable, juicier and flavorful compared to other imported beef breeds is its high marbling or intramuscular fat level [3]. Because, marbling has been proven to positively affect eating quality attributes such as tenderness, flavor and juiciness [4]. It is well recognized that eating quality attributes especially tenderness and flavor are extremely important factors determining purchasing decision by consumers for beef in many meat markets [5]. A recent study by O’Quinn et al [6] also reported that tenderness and flavor become the most important drivers of beef eating satisfaction. In Korea, studies regarding the consumer’s choice have shown that Korean consumers much prefer Hanwoo beef and they are willing to pay more money for the more abundantly marbled-Hanwoo beef because it is regarded as a premium product [7]. In the Korea meat industry, 24 h after slaughter, all cattle carcasses are evaluated for meat quality grade by official graders using Korean carcass grading system (KAPE) [8], and five different quality grades such as; 1^++^, 1^+^, 1, 2, and 3 are classified, mainly depending on the degree of marbling and other parameters such as meat and fat color [9]. As a result of Korean consumers’ preference, the average prices for the higher quality grades such as; grade 1^+^ and 1^++^ loin cuts are about 54 and 64 US dollars which are several times higher than the average prices (24 US dollars/kg) for those from the lower quality grade groups (e. g., grade 2 and 3) [8] The Hanwoo beef cuts from low quality grade group (e.g., grade 3) are not only cheaper in price but also of redundancy. Additionally, the beef cuts with low quality grade are often sold at a discount. This is, in part because the beef cuts with low quality grade are tougher and less flavorful [10]. In order to deal with the problem, solutions include; applying post-mortem ageing methods or/using suitable methods of cooking. In meat industry, post-mortem ageing is the most commonly-applied method to enhance tenderness and flavor of beef [11].

Sous vide, initially developed in France since 1970s, is a method of cooking that involves sealing the food in plastic bag and placing it in a water bath at a specific temperature (50°C to 70°C) for an extended period of time (several hours to multiple days) [12]. This cooking method allows for greater control and consistency to achieve higher uniformity in degree of doneness [13]. In meat cooking, the sous vide technique has become a method to produce good textural properties (e.g., tender and juicier) and better sensory quality, and minimizing shrinkage of meat [13,14]. Furthermore, sous vide is one of the cooking methods effectively increasing the low value of tough meat cuts by transforming into tender meat cuts [15,16]. Thanks to these advantages, perhaps, sous vide is widely known and extensively used in restaurants and by foodies over the world in recent decades [16,17]

Apart from these advantageous points, however, the sous vide cooking method also has some drawbacks such as; the sous vide-cooked meat usually lacks browning on the surface and roasted aroma characteristics [16]. It is well known that the development of the roasted aroma and surface brown color in meat during cooking results from the Maillard reaction between amino acids and reducing sugar, and lipid oxidation [18]. Therefore, the lack of surface brown color and roasted aroma notes in the sous vide-cooked meat probably is due to the low temperatures (around 60°C to 80°C) and less dehydration on the meat surface [18]. In order to overcome this problem, chefs frequently roast or fry the surface of sous vide-cooked meats [17]. More recently, researchers have coupled the sous vide cooking with roasting (sous vide followed by roasting or roasting before sous vide) and reported that roasting either before or after sous vide results in lamb meat with a browner surface and a more intense cooked meat flavor [16].

Searing with a blowtorch is one of the best way to enhance the brown and roasted flavor on the outside of a beef steak. This is due to the chemical reactions such as Maillard reaction that takes place when amino acids and sugar are heated to a high temperature [19] Though the blowtorching and sous vide method has been widely used in cooking beef [15], with the best of our knowledge, no attention has been paid to investigating the combined effects of sous vide cooking with roasting/or blowtorching on the final eating quality of beef. We hypothesized that roasting and blowtorching with their extremely different heat levels may produce various effects on the Maillard reactions (e.g., browning and volatile aroma compounds) which subsequently affects the color and flavor characteristics of sous vide cooked meat. Therefore, the objective of this study was to investigate the combined effects of postmortem ageing and sous vide cooking followed by roasting/or blowtorching on the eating quality properties and volatile flavor compounds of low quality Hanwoo beef cut.

## MATERIALS AND METHODS

### Beef cut samples

Beef striploins (n = 15) obtained from carcasses (low-quality grade 3) of Hanwoo steers (36 months old) after 24 h slaughter were used in the present investigation. Each beef striploin was vacuum-packaged individually in plastic bags, kept in cooler box and transported to the Laboratory of Meat Science, Chonbuk National University for analysis.

### Postmortem ageing treatment

For chiller ageing treatment, each of the striploins were divided into two equal portions (a total of 30 portions were made from the 15 striploins), and a half (n = 15; 7 from cranial and 8 from caudal portions) were assigned to the 0-day ageing while, the remaining portions (n = 15) were assigned to the 14-day ageing treatment. The 0-day aged samples were immediately used for analyses (on the sampling day) while, the samples assigned to the 14-day ageing were individually vacuum-packaged in a nylon/PE vacuum bag, and then aged in an ageing room at 4°C.

### Sous vide cooking

When the ageing was completed, all the 0 and 14 d-aged samples were subjected to sous vide cooking treatment. For the sous vide cooking, each aged sample was made into 2.5-cm thick steaks (30 steaks per ageing group) which were then 80% vacuum-packaged individually in plastic bags (nylon/polyethylene pouches). The steaks were sous vide cooked at 55°C for 5 h and then raised to 60°C for an additional 1 h in a temperature controlled water bath as described in our previous study [20]. Once the cooking was completed, the cooked samples were removed from the water bath and cooled for 30 min in a circulatory water bath and then chilled overnight in a chilling room.

### Roasting and blowtorch treatment of sous vide cooked-steaks

In order to achieve the sous vide-cooked beef steaks with brown color on their surfaces as well as attractive roasted flavors, these samples were subjected to additional cooking treatment such as roasting and blowtorching. Out of the 30 sous vide- cooked steaks (in each ageing group), ten of those were considered as control, another ten were roasted at 250°C for 20 min in an oven with dry air (SV+OV) [16], and the rest of the steaks (n = 10) were blowtorched for 2 min on both sides of the steaks (1 min per side) (SV+TC). Thereafter, each 7 steaks from the sous vide cooking (SV), SV+OV, and SV+TC at each ageing period were used for volatile flavor compounds analysis and sensory evaluation, while the remaining 3 steaks were placed in plastic bags, sealed and stored overnight at 4°C for microbial analysis and instrumental color measurement.

### Wanrner-Bratzler shear force value measurement

In order to determine whether the ageing and sous vide cooking enhances tenderness of the beef samples, Wanrner-Bratzler shear force (WBSF) was measured on the steaks cooked to a standard core temperature of 70°C (control) as recommended by American Meat Science Association (AMSA) [21] and those cooked by the sous vide method. The WBSF was measured on cores (5 cores/steak) with an average diameter of 0.5 inches removed parallel to the muscle fiber direction using a 0.5-inch metal corer. The WBSF values were obtained by completely cutting the cores in an Instron Universal Testing Machine (Model 3342, Instron Corp, Norwood, MA, USA) using a V-shaped WB blade at a crosshead speed of 200 mm/min and a 40 kg load cell.

### Microbial analysis

Raw beef before and after sous vide cooking (after chilling overnight) was used for microbial analysis. Immediately after removing the plastic bags, microbiological samples were aseptically taken by swabbing on 10 cm^2^ surface of sample, transferred to sterile plastic tubes containing 10 mL of peptone water. After vortexing, serial dilutions were then made in the peptone water. Aerobic bacteria were determined by spreading 1mL of appropriate dilutions onto 3M Petrifilm Aerobic Count Plate (3M Health Care., Paul, MN, USA) while, lactic acid bacteria (LAB) were determined by spreading 0.1 mL of appropriate dilutions onto Man Rogosa Sharpe (MRS) agar plates. The plates were then incubated for 24 h at 37°C. Each sample was done in duplicates and total aerobic bacteria as well as LAB were expressed as log_10_ numbers of colony forming units/gram (cfu/cm^2^).

### Instrumental color traits and browning index measurement

The instrumental color traits and browning index (BI) were determined at 4 different spots on the surface of each using steak Konica Minolta Spectrophotometer CM-2500d with an 8 mm measuring port, D 65 illuminant and 10° observer. Hunter L (lightness), a (redness), b (yellowness) values were recorded. The data presented are means of four measurements. The BI was calculated using the above obtained Hunter L*, a*, and b* values: BI = (100×[x−0.31])/0.17; where: x = ([a*+1.75×L*])/([5.645×L*+a*−3.012×b*]), as Jorge et al [16].

### Sensory evaluation

The SV, or SV+OV and SV+TC striploin steaks were assessed by a 12-member untrained panel using a descriptive analysis method following the methods of Deda et al [22] and Meilgaard et al [23] with suitable modifications. The member panels were chosen from students, researchers and faculty at the university. Prior to the assessment, the samples were warmed up for 1 min in a microwave. Thereafter, each the steak was prepared into 6 slices (40 mm×40 mm×4 mm) which were then assessed by 6 panelists. The slices were dispensed on individual plates and served to the panelists. The samples were evaluated for flavor, juiciness, tenderness and overall acceptability using a 7-point hedonic scale. In which: tenderness (7 = extremely tender, 6 = very tender, 5 = moderately tender, 4 = neither tender nor tough, 3 = moderately tough, 2 = very tough, and 1 = extremely tough); juiciness (7 = extremely juicy, 6 = very juicy, 5 = moderately juicy, 4 = neither juicy nor dry, 3 = moderately dry, 2 = very dry and 1 = extremely dry); flavor and acceptability (7 = like extremely, 6 = very like, 5 = moderately like, 4 = neither like nor dislike, 3 = moderately dislike, 2 = very dislike, 1 = dislike extremely). Drinking distilled water and salt-free crackers were provided between samples to cleanse the palate. All sensory sessions were carried out in the sensory panel booth room equipped with white lighting.

### Volatile flavor compound identification

Following the sensory evaluation, the remaining samples were immediately used for analysis of flavor compounds. The component of volatile flavor compounds was analyzed by solid phase micro-extraction (SPME) following the procedure of Ba et al [24]. Briefly, about 1.0 g of sample was taken and placed into a 20-mL headspace vial (Part No. 5188-2753, Agilent, Santa Clara, CA, USA) and 1.0 μL of internal standard (2-methyl-3-heptanone, 816 mg/mL in methanol) was also added. The vial was then sealed with PTFE-faced silicone septum for extraction. A SPME device containing carboxen–polydimethylsiloxane (75 μm) fiber (Supelco, Bellefonte, PA, USA) was used for extraction of the compounds. All steps such as; extraction, absorption, desorption of the flavor compounds were done using a fully automated SPME sample preparation instrument (Model: AOC-5000 Plus) connected to gas chromatography (GC) (Model: 7890B GC) with mass spectrophotometry (MS) (Model: 5977B MSD, Agilent Technologies, USA). The extraction was carried out at 65°C and agitated at 250×rpm for 60 min. Volatiles were separated on a DB-5MS capillary column (30 m×0.25 mm i.d.×0.25 μm film thickness, Agilent J & W Scientific, Model No.122-5532, Folsom, CA, USA). The GC/MS conditions set were same as those by Ba et al [25]. Compounds were identified by comparing their mass spectra with those already present in the Wiley registry of mass spectral data (Agilent Technologies, USA) and by comparing their retention times with those of external standards. Approximated quantities of the volatile compounds were quantified by comparison of their peak areas with that of the internal standard obtained from the total ion chromatogram using a response factor of 1. Each steak sample was analyzed in duplicates.

### Statistical analysis

The Statistic Analysis System (SAS) package (SAS Institute, Cary, NC, USA; 2007) was applied for analyzing the obtained data. Means and standard errors were calculated for the variables. The data were analyzed by using the general linear model procedure considering ageing and cooking as the main effects. Means were compared using Duncan’s multiple range test. The correlations between the cooking treatments (SV, SV+OV, and SV+TC) with quality parameters, volatile flavor compounds and sensorial traits were determined using Pearson’s linear correlation coefficient. The significance was defined at p<0.05.

## RESULTS AND DISCUSSION

### Effect of ageing and cooking treatment on WBSF and microbial quality

Table 1 shows the WBSF values of striploin steaks cooked to core temperature of 72°C (a standard core temperature used to determine shear force as recommended by American Meat Science Association [21]) and those cooked by the sous vide method, as well as the microbial count of steaks before and after sous vide cooking. Till now, WBSF is the most widely used instrumental method to evaluate meat tenderness, and its results are good predictions of tenderness ratings. Basically, the higher WBSF values obtained from meat are negatively related to consumer perception of tenderness and overall acceptance [26]. Results showed that the WBSF value (55.30 and 35.50 N after 0 and 14 d ageing, respectively) of striploin steaks cooked to the core temperature of 72°C (standard) were significantly higher than the values (42.26 and 23.43 N after 0 and 14 d ageing, respectively) of those cooked by sous vide method (p<0.05). It was estimated that after the sous vide cooking the shear force of steaks was reduced by nearly a half. Our results are in good agreement with the finding of Ismail et al [15]. Ageing significantly reduced the WBSF of the samples regardless of cooking method (70°C cooking or sous vide), however, it was observed that ageing for 14 d followed by sous vide cooking produced the steaks with the lowest shear force (23.43 N). Especially, when compared to the shear force values (27–45 N) of *semitendinosus* muscle steaks (from same cattle breed and same quality grade) cooked with sous vide under different durations and temperatures [15], all the striploin steaks cooked by sous vide method had considerably lower value after 14 d ageing. This implies that the combination of postmortem ageing and sous vide cooking could effectively improve tenderness of the low quality grade beef-derived steaks. Researchers have found that meat toughness increases when cooked at temperature ranges of 40°C to 50°C and 60°C to 80°C whereas, it decreases between 50°C and 60°C [27]. In the present study, the steak samples were sous vide- cooked at 55°C to 60°C, and the decrease in WBSF values may be due to the denaturation of perimysium connective tissue and collagen shrinkage [15]. On the other hand, previous studies reported that sous vide-processed products generally exhibit a highly microbial growth during storage [28]. In the present study (as displayed in Table 1), total aerobic plate count (APC) and LAB for the raw steaks (before sous vide cooking) at 0 and 14 d ageing were found at 2.39 and 1.16 (log_10_ CFU/cm^2^) and 4.71 and 2.30 (log_10_ CFU/cm^2^), respectively. Noticeably, no APC or LAB growth was detectable in the sous vide-cooked steaks, meaning that under the sous vide cooking conditions employed in this study, all the microbes were eliminated, which agrees with the finding of Jeong et al [29].

### Effects of ageing and cooking treatment on color properties

The effects of sous vide cooking and/or following by oven roasting or blowtorching on the color parameters of striploin steaks are presented in Table 2. It was observed that roasting in oven at 250°C for 20 min or blowtorching for 2 min after sous vide significantly affected the values of color parameters. In the current study, the sous vide-cooked steaks exhibited a lighter color (L* = 44.62 and 44.44, at 0 and 14 d ageing, respectively) compared to those further treated by roasting (28.64 and 24.32 at 0 and 14 d ageing, respectively) and blowtorching (24.02 and 24.13 at 0 and 14 d ageing, respectively). The higher L* value could be due to the higher level of exuded water remains on the surface of sous vide-cooked steaks [30]. The ageing did not affect the L* values of any of the samples (p>0.05), except for the SV+OV samples (p<0.05). The L* values of the sous vide-cooked steaks in the present study were similar to values reported for beef samples cooked at same temperature (60°C) by Roldan et al [31]. Contrastingly, Ismail et al [15] reported higher L* values (58.16) for semitendinosus muscle steaks sous vide-cooked at 60°C for 6 h. These contrasting results could be related to the differences in muscle type, gender and animal age etc. Sous vide cooking followed by blowtorching led to significantly higher redness values compared to the steaks which were oven roasted after sous vide cooking or just sous vide cooking (p<0.05). While, the sous vide-cooked steaks exhibited higher yellowness values compared to the SV+OV and SV+TC samples on both days examined (p<0.05).

It is recognized that cooking meats with sous vide generally has some drawbacks such as, the lack of surface brown color that is not attractive to consumers. In order to counteract this problem, in the present study, after sous vide cooking the steaks were subjected to high temperatures treatment such as roasting in oven or blowtorching. Results revealed that blowtorching after sous vide (SV+TC) significantly increased the BI values on the surface compared to the sous vide (SV) or sous vide followed by oven roasting (SV+OV) (p<0.05). Similar observation reported by Jorge et al [16] for lamb loin sous vide cooked for 12 h followed by roasting for 15 min at high temperature. Researchers have proven that the brown color on meat’s surface results from formation of colored compounds named melanoidins through the Maillard reaction [32]. The mechanism underlying the increased BI values may be related to the more intense dehydration of the steak’s surface which favors for the formation of brown compounds. Interestingly, postmortem ageing was found to increase the BI values; after 14 d ageing all the sous vide- cooked samples or those followed by roasting and blowtorching had significantly higher BI values compared to those aged for 0 d (p< 0.05). This result could be explained due to the increased release of meat juice to the meat surface during postmortem ageing as a result of proteolytic activity, since this juice contains a huge amount of flavor precursors (e.g., free amino acids and reducing sugars) which subsequently participate in the Maillard reaction (browning reaction) at high temperature (blowtorching) to produce the colored compound as well as aroma compounds. In accordance with the above described results, the result of Pearson’s correlation (Table 3) also shows that the sous vide-cooked samples were positively correlated to lightness (r = 0.994) and negatively correlated to BI (r = −0.900). While, the sous vide-cooked samples followed by blowtorching (SV+TC) were positively correlated to BI (r = 0.969) and negatively correlated to lightness (r = 0.967).

### Effects of ageing and cooking treatment on volatile flavor compounds

Volatile flavor compounds mainly responsible for the development of aromas of cooked meat are formed from the precursors (e.g., amino acids, reducing sugars and fatty acids etc.) in meat during cooking/heating [33]. Till now, thousands of volatile compounds have been identified from various cooked meat types and many out of them have been reported to significantly contribute to flavor characteristics of cooked meat [34]. It was proven that the three main routines responsible for producing the volatile compounds in cooked meats are i) Maillard reaction (a chemical reaction between amino acids and reducing sugars), ii) lipid oxidation/degradation, and iii) the interaction between the lipid-degraded products with Maillard reaction products [34]. Noticeably, the quality and quantity of the volatile compounds generated strongly depend upon the level of heat (vary among the cooking methods) applied on the meats for instance; the Maillard reaction is intensified at above 140°C (e.g., roasting and grilling) while, the lipids-oxidized and degraded products are more predominant at lower cooking temperature such as in boiled and steamed mea products [33,34]. The effects of ageing and cooking treatments on the number and quantity of volatile flavor compounds are summarized in Table 4. The outcome of our analysis revealed that a total of 48 volatile compounds were tentatively detected and identified in the Hanwoo beef striploin steaks sous vide (SV) cooked or SV followed by oven roasting (SV+OV) and followed by blowtorching (SV+TC). Based on their chemical families, these compounds were classified into the following classes: aldehydes (17), alcohols (8), pyrazines (5), sulfur-containing compounds (5), furans (2), and hydrocarbons (11).

Aldehydes were the most predominant class found in this study, which agrees well with the finding of Kim et al [35] and Ba et al [25]. Previous studies have reported that aldehydes associated with green, fatty and fruity odor notes at very low odor detection threshold are important in the development of flavor characteristics cooked meat [34,36]. Six out of 17 aldehydes were significantly affected by the cooking treatment (p<0.05). Amongst, 2-methyl butanal and 3 methyl butanal are the Strecker degradation (a part of Maillard reaction) products of leucine and isoleucine, respectively [37]. The amounts of these two compounds were higher in the SV+TC-treated samples compared to those treated with SV+OV (p<0.05) whereas, they were not detectable in the sous vide-cooked samples. This implies that the Maillard reaction of the amino acids was enhanced due to the increased temperature during cooking treatment. Furthermore, benzaldehyde (the oxidized product of C18:3n-3), and octanal (the oxidized product of C18:1n-9) [38], whose amounts were also significantly higher in the samples treated with the SV+TC compared to those just sous vide cooked (p<0.05). Octanal is an important compound that contributes to the fruity-fatty and oily odor notes of cooked meat [39]. The postmortem ageing apparently showed a minor effect on the aldehydes; only nonanal decreased whereas, E-nonenal increased its amount with increased ageing time up to 14 d (p<0.05). Additionally, the cooking treatment (e.g., blowtorching) considerably affected the total amount of aldehyde class (Table 5). The total aldehydes were significantly higher in the samples treated with SV+TC compared to those just sous vide cooked (p<0.05) (Table 5). These results could be explained due to the two steps of cooking treatment: the sous vide cooking leads to the formation of lipid oxidation-derived aldehydes and free radicals which favor the rate of aldehydes production from the lipid oxidation reactions during further roasting and blowtorching treatment [16]. In line with these observations, the result of Pearson’s correlation analysis (Table 3) also show that the samples treated with SV+TC were positively correlated to 2-methyl butanal (r = 0.956), 2-methyl butanal (r = 0.897), and Nonanal (r = 0.891) (p<0.05). This means that increasing the cooking temperature for instance; by using oven roasting at 250°C or blowtorching after the sous vide cooking favored the generation of these aldehydes which are associated with the pleasant odor notes as mentioned above.

Alcohols have been reported to contribute to cooked meat flavors due to their low odor-detection threshold [34]. The alcohols were the third most predominant class of volatiles, with 8 compounds were found in the samples at 0 and 14 d ageing. Most of these alcohols have been identified and reported for cooked meats in literature [25,34,40]. Results showed that only 1-octen-3-ol (the product of C18:2n-6 oxidation) showed statistical difference among the treatments, with its higher amount being found in the samples treated with SV+ OV or SV+TC compared to those just sous vide cooked (p< 0.05). Two interesting exceptions were 1-octanol and 1-undecanol, which were not found in the sous vide-cooked samples, but were found in the samples roasted or blowtorched after the sous vide cooking.

Pyrazines, the Maillard reaction-derived products have been reported to be associated with pleasant odor notes (e.g., roasty) which significantly contribute to the cooked meat flavor [34,36]. It seems that the most interesting result obtained in the volatiles analysis was the pyrazine class. Particularly, only one compound (3-ethyl-2,3-methyl pyrazine) was found in the SV-cooked samples while, all the five identified pyrazines were found in the samples sous vide cooked followed by oven roasting (SV+OV) or blowtorching (SV+TC). Especially, the amounts of most of pyrazines such as 3-ethyl-2,5-dimethyl pyrazine and trimethyl pyrazine were significantly higher in the samples treated with SV+TC compared to those treated with SV+OV (p<0.05). Similarly, previous studies have reported that pyrazines are the major class of volatile compounds in meats grilled at high temperature [18,34]. The ageing led to an increase in level of 3-ethyl-2,3-methyl pyrazine in the samples treated with SV+OV and SV+TC (p< 0.05). Furthermore, the total amount of pyrazines were significantly higher the samples treated with SV+TC followed by the SV+OV and SV on both ageing days (p<0.05) (Table 5). As far as the pyrazines are important, the amount of the pyrazines increased in the oven roasting or blowtorching after sous vide, such increases may be the consequence of more intense Maillard reaction due to the high heating treatment. In contrast, only one pyrazine found in the SV- cooked samples as mentioned above, probably is due to the low cooking temperature (55°C to 60°C) and higher moisture content of the surface that inhibits pyrazines production by the Maillard reaction. The result of Pearson’s correlation analysis (Table 3) also revealed that the SV+TC- treated samples were highly correlated to Methyl pyrazine (r = 0.896), 3-ethyl-2,5-dimethyl-pyrazine (r = 0.981), Trimethyl pyrazine (r = 0.936) and total pyrazines (r = 0.891) (p<0.05).

Sulfur-containing heterocyclic compounds are known to be the products derived from the Maillard reaction or the Strecker of sulfur-containing amino acids. The sulfur-containing compounds associated with roasty, meaty and onion odor notes play a key role in the cooked meat flavors [19,34]. Results showed that only three (carbon disulfide, 2-thiophene methanol and 3-phenyl thiophene) out of the five sulfur-containing compounds were found in the SV-cooked samples whereas, all of them were found in the samples treated with SV+OV and SV+TC. No statistical differences occurred among the SV, SV+OV and SV+TC for all the compounds (p>0.05). In general, most of these sulfur-containing compounds have been reported for cooked meats [25,40]. The ageing apparently had a minor effect on the sulfur-containing compounds; 2-thiophen methanol increased in its amount, and 3-phenyl thiophene decreased in its amount in the samples treated with the SV+TC and SV+OV, respectively after 14 d ageing (p<0.05). The reason behind the increased amounts of these two compounds could be related to increased levels of flavor precursors (sulfur-containing amino acids: cysteine and methionine) resulting from the proteolysis during the postmortem ageing.

Though the amount of individual sulfur-containing compounds showed no statistical difference as mentioned above, the total amount of this class was significantly higher in the samples treated with the SV+OV or SV+TC compared to those just SV cooked (p<0.05) (Table 5). Thus, the oven roasting or blowtorching treatment after the sous vide partly stimulated the generation of sulfur-containing compounds, thus probably enhancing the intense flavor characteristics of sous vide – cooked beef.

Finally, furans and hydrocarbons are mainly derived from the oxidation of lipids such as C18:2n-6 and C18:3n-3 [34,38]. These volatile classes possess a high odor detection threshold, thus contributing less significantly to flavor development of cooked meat [34]. In the present study, hydrocarbons were the second most predominant class (11 compounds) found in the samples. However, only three compounds (3,4-dimethyl heptane, 2-methyl heptane and hexadecane) showed statistical difference among the treatments (p<0.05). It may be said that the cooking treatment (e.g., roasting or blowtorching after sous vide) and ageing had a minor effect on the components of the hydrocarbons class. This is in agreement with result reported by Roldan et al [18] for sous vide-cooked lamb loin.

Overall, it seems then clear that the sous vide cooking at low temperature (55°C to 60°C) resulting in a lack of surface dehydration leads to a smaller quantity of the volatile compounds derived from Maillard reactions in the cooked beef steaks. However, this drawback could be overcome by combining sous cooking with other more intense heat treatment such as roasting in oven or blowtorching. As a consequence, these thermal treatment processes boosted the Maillard reaction on the surface, thus promoting the generation of Maillard reaction-derived flavor compounds such as pyrazines and sulfur-containing compounds which subsequently may enhance the roasty and meaty intensity of cooked beef.

### Effects of ageing and cooking treatment on eating quality properties

Satisfaction is an important factor determining the quantity of meat that is purchased by consumers [5,6], and the most important aspect of meat quality is eating quality, usually defined as scores given by taste panelists. The effects of ageing and cooking treatment on the sensorial traits are presented in Table 6. As expected, the flavor scores given by the panelists were significantly higher in the samples treated with sous vide + blowtorching (SV+TC), followed by sous vide + oven roasting (SV+OV), and the lowest in the samples just sous vide cooked (p<0.05). The results indicating the higher flavor scores for the samples roasted or blowtorched after sous vide cooking may be explained due to the more intense cooked flavor caused by higher amount of distinct flavor compounds such as pyrazines and sulfur-containing compounds (Tables 4, 5). On the other hand, the results of Pearson’s correlation analysis also revealed that the flavor score was positively correlated to Methyl pyrazine (r = 0.985), 2,4-dimethyl pyrazine (r = 0.916), 3-ethyl-2,5-dimethyl pyrazine (r = 0.987), trimethyl pyrazine (r = 0.963) and 2-methylthiophene (r = 0.867) (data not shown). This implies that the flavor intensity of cooked beef increased with increased concentrations of these flavor compounds.

Regarding juiciness, blowtorching after sous vide cooking (SV+TC) did not show a negative effect on the juiciness compared to the samples just sous vide cooked, as no statistical difference in juiciness scores occurred between the SV and SV+TC (p>0.05). Unexpectedly, the samples roasted in oven after sous vide cooking exhibited a drier appearance, indicated by a significantly lower juiciness score compared to those just sous vide cooked or blowtorched after sous vide (p<0.05). This result could be related to the excess dehydration or moisture loss caused by roasting in oven for a long duration (20 min). Our result is in agreement with finding reported by Jorge et al [16] for lamb loin. Additionally, the Pearson’s correlation analysis also revealed that the samples roasted after sous vide cooking was negatively correlated to the juiciness score (r = −0.985).

A similar trend as observed on the juiciness was found for the tenderness in which the samples roasted in oven after the sous vide cooking (SV+OV) presented a significantly lower tenderness score compared to those just sous vide cooked or sous vide cooked followed by blowtorching (SV+TC) (p< 0.05). This result could be due to a higher level of moisture loss caused during roasting in oven, which made the samples become drier and tougher. Interesting, the panelists gave similar tenderness scores for the SV and SV+TC samples (p>0.05). Regarding the overall acceptance, the panelists gave the highest score for the samples treated with SV+TC, followed by the SV+OV and SV on both examining days (0 and 14 d). This could be explained due to association with the synergistic effect of their higher tenderness, juiciness and especially flavor scores. Noticeably, postmortem ageing also increased the overall acceptability scores of the sous vide-cooked and the SV+TC-treated samples.

## CONCLUSION

The present study for the first time, evaluated the combined effects of postmortem ageing and cooking treatments (sous vide alone, sous vide followed by oven roasting or blowtorching) on the quality properties of low quality grade Hanwoo beef striploin. In general, the sous vide cooking of beef resulted in significantly lower WBSF and safety in term of microbiology. Blowtorching after sous vide cooking produced a browner surface of beef steaks. Particularly, roasting or blowtorching after sous vide cooking resulted in higher quantities of important aroma flavor compounds such as pyrazines and sulfur-containing compounds compared to those just sous vide cooked. Furthermore, roasting or blowtorching after sous vide cooking resulted in increased flavor scores of cooked beef steaks. However, compared to the roasting in oven the blowtorching after sous vide cooking seemed to produce more beneficial effects on the improvement of the technological quality (e.g., redness and BI) and eating quality (e.g., flavor intensity), and especially juiciness maintenance of cooked beef. Postmortem ageing beef for 14 d significantly improved the tenderness by reducing the WBSF values, and increasing the sensorial tenderness scores. Based on the results obtained in the present study, it may be concluded that the combination of postmortem ageing and sous vide cooking followed by blowtorching or oven roasting should be applied to improve the eating quality attributes such as tenderness and flavor as well as the overall acceptability of the low quality grade Hanwoo beef.

## Figures and Tables

**Table 1:** Shear force value and microbial count of sous vide-cooked beef striploin steaks

Items	Ageing (d)	Control	Sous vide cooking
Wanrner- Bratzler Shear force (N)*	0	55.30±3.1^aA^	35.50±1.60^bA^
	14	42.26±3.22^aB^	23.43±1.72^bB^
Total plate count (log_10_/cm^2^)**	0	2.39±0.14^B^	nd
	14	4.71±0.50^A^	nd
Lactic acid bacteria (log_10_/cm^2^)**	0	1.16±0.11^B^	nd
	14	2.30±0.14^A^	nd

* The shear force was determined on the beef steaks which were cooked to a core temperature of 70°C as recommended by American Meat Science Association (AMSA, 2015).

** The bacterial load was determined on the fresh striploin steaks (control) or sous vide-cooked steak samples at the end of each ageing period.

Means within a same row with different superscripts (^a,b^) differ significantly at (p<0.05).

Means within a same column in each item with different superscripts (^A,B^) differ significantly at (p<0.05); nd, not detectable.

**Table 2:** Color traits of beef striploin steaks as affected by ageing and cooking treatment

Items	Ageing (d)	SV1)	SV+OV1)	SV+TC1)
Lightness	0	44.62±0.82^a^	28.64±0.14^Ab^	24.02±0.95^c^
	14	44.44±0.52^a^	24.32±0.22^Bb^	24.13±0.10^b^
Redness	0	7.15±0.30^Ab^	5.84±0.29^c^	8.93±0.68^Aa^
	14	5.42±0.15^Bb^	5.23±0.51^b^	7.84±0.11^Ba^
Yellowness	0	13.26±0.10^Ba^	7.55±0.60^Bc^	9.02±0.28^b^
	14	16.21±0.11^Aa^	9.47±0.15^Ab^	7.80±0.37^c^
Browning index	0	46.52±1.05^Bb^	45.03±0.16^Bb^	62.25±1.12^Ba^
	14	53.58±1.16^Ab^	64.29±0.24^Ab^	73.58±4.80^Aa^

1) SV, sous vide cooking at 55°C for 5 h and raised to 60°C for 1 h; SV+OV, sous vide cooking followed by oven roasting at 250°C for 20 min; SV+TC, sous vide cooking followed by blowtorching for 2 min.

Means within a same row with different superscripts (^a–c^) differ significantly at (p<0.05).

Means within a same column in each parameter with different superscripts (^A,B^) differ significantly at (p<0.05).

**Table 3:** Correlation coefficients between the color traits, flavor compounds and eating properties with various cooking treatment

Items	SV1)	SV+OV1)	SV+TC1)
Lightness	0.994*	−0.406	−0.858*
Redness	−0.264	−0.703	0.967*
Yellowness	1.000*	−0.488	−0.512
Browning Index	−0.900*	−0.269	0.969*
Butanal, 2-methyl-	−0.731	−0.225	0.956*
Butanal, 3-methyl-	−0.766	0.866	0.897*
Hexanal	−0.139	0.927*	−0.788
heptanal	−0.605	−0.387	0.692
E-2-heptenal	−0.500	1.000	−0.500
Benzaldehyde	−0.955*	0.735	0.220
Octanal	−0.929	0.143	0.786
Benzenacetaldehyde	−0.629	−0.359	0.688
Nonanal	−0.912*	0.101	0.891*
E-2-octenal	−0.989	0.623	0.365
E-2-nonenal	−1.000	0.500	0.500
E,4-decenal	1.000	−0.500	−0.500
E,2-decenal	0.189	−0.945	0.756
1-pentanol	0.809	0.104	−0.913*
1-heptanol	0.500	−1.000	0.500
1-octen-3-ol	−0.971*	0.693	0.277
2-nonen-1-ol	0.945*	−0.189	−0.756
1-octanol	−0.988	0.629	0.359
1-Hexanol	1.000	−0.500	−0.500
Pyrazine, methyl-	−0.832	−0.064	0.896*
Pyrazine, 2,4-dimethyl-	−1.000*	0.500	0.500
Pyrazine, 3-ethyl-2,5-dimethyl-	−0.839	−0.052	0.981*
Pyrazine,2,3-dimethyl-	−0.961*	0.721	0.240
Trimethyl pyrazine	−0.773	−0.163	0.936*
2-methylthiophene	−0.992	0.603	0.390
2-thiophene methanol	−0.500	1.000	−0.500
3-thiophene-carboxaldehyde	−0.756	0.945	−0.189
3-phenyl thiophene	0.756	−0.945	0.189
2-pentylfuran	1.000*	−0.500	−0.500
2-n-octylfuran	−0.971	0.693	0.277
Toluene	0.500	−1.000*	0.500
2-heptanone	−0.500	1.000*	−0.500
Propanal	−0.756	0.945	−0.189
Propanal, 2-methyl-	−0.971	0.277	0.693
Butanal	−0.866	0.000	0.866
Butanal, 3-methyl-	−0.832	−0.064	0.986*
Flavor	−0.916*	0.110	0.896*
Juiciness	0.642	−0.985*	0.343
Tenderness	0.374	−0.990*	0.616
Overall acceptability	−0.723	−0.237	0.960
Total aldehydes	−0.952*	0.742	0.209
Total alcohols	0.217	0.737	−0.954
Total pyrazines	−0.919*	0.118	0.891*
Total sulfur-containing compounds	−0.950*	0.745	0.205
Total furans	0.997*	−0.434	−0.564
Total hydrocarbons	1.000*	−0.500	−0.500

1) SV, sous vide cooking at 55°C for 5 h and raised to 60°C for 1 h; SV+OV, sous vide cooking followed by oven roasting at 250°C for 20 min; SV+TC, sous vide cooking followed by blowtorching for 2 min.

* Significant at p<0.05.

**Table 4:** Concentration (μg/g) of volatile flavor compounds in beef striploin as affected by ageing and cooking treatment

Compounds	Retention time	IM1)	Ageing (d)	SV2)	SV+OV2)	SV+TC2)
Aldehydes						
Propanal	1.701	MS+	0	0.02±0.001	0.02±0.001	0.01±0.001
		STD	14	0.01±0.001	0.03±0.002	0.02±0.001
Propanal, 2-methyl-	1.942	MS+	0	nd	0.01±0.001	0.01±0.001
		STD	14	nd	0.02±0.002	0.03±0.001
Butanal	2.142	MS+	0	0.02±0.001	0.02±0.001	0.02±0.001
		STD	14	0.02±0.001	0.04±0.003	0.06±0.002
Butanal, 3-methyl-	2.703	MS+	0	nd	0.03±0.001	0.01±0.001
		STD	14	nd	0.05±0.01b	0.17±0.03^a^
Butanal, 2-methyl-	2.817	MS+	0	nd	0.02±0.001	0.03±0.002
		STD	14	nd	0.07±0.009^b^	0.27±0.01^a^
2-methyl-heptanal	3.292	MS+	0	0.12±0.01	0.09±0.02	0.08±0.005
		STD	14	0.07±0.002	0.13±0.002	0.08±0.002
Hexanal	6.09	MS+	0	1.77±0.12	1.87±0.06	1.27±0.01
		STD	14	1.04±0.02	1.94±0.008	0.93±0.02
Heptanal	9.26	MS+	0	0.84±0.05	0.80±0.007	1.30±0.02
		STD	14	0.22±0.01	0.32±0.002	0.20±0.009
E-2-heptenal	10.748	MS+	0	0.02±0.002	0.03±0.001	0.01±0.001
		STD	14	0.01±0.001	0.01±0.001	0.02±0.001
Benzaldehyde	10.868	MS+	0	0.19±0.01	0.37±0.04	0.20±0.008
		STD	14	0.08±0.002^b^	0.13±0.008^ab^	0.23±0.01^a^
Octanal	11.921	MS+	0	0.39±0.02^b^	0.65±0.001^a^	0.61±0.03^a^
		STD	14	0.17±0.02^b^	0.23±0.004^ab^	0.32±0.03^a^
Benzenacetaldehyde	12.877	MS+	0	0.02±0.001	0.02±0.001	0.01±0.001
		STD	14	nd	0.02±0.003	0.06±0.006
Nonanal	14.199	MS+	0	0.53±0.005^b^	0.77±0.006^abA^	0.86±0.02^aA^
		STD	14	0.33±0.01^c^	0.43±0.001^Bb^	0.51±0.03^Ba^
E-2-octenal	13.185	MS +	0	0.03±0.001	0.02±0.009	0.03±0.001
		STD	14	0.03±0.001	0.02±0.002	0.02±0.002
E-2-nonenal	15.329	MS +	0	0.03±0.002	0.06±0.001^A^	0.06±0.001^A^
		STD	14	nd	0.01±0.001^B^	0.01±0.001^B^
E,4-decenal	15.999	MS	0	0.08±0.001^a^	0.01±0.001^b^	0.01±0.001^b^
			14	nd	0.01±0.001	0.01±0.001
E,2-decenal	17.288	MS+	0	0.18±0.001^a^	0.03±0.002^Bb^	0.07±0.06^ab^
		STD	14	0.03±0.002^b^	0.10±0.004^Aab^	0.18±0.01^a^
Alcohols						
1-penten-3-ol	3.046	MS+	0	nd	0.005±0.001	0.01±0.001
		STD	14	nd	0.01±0.001	0.01±0.001
1-pentanol	5.015	MS+	0	0.18±0.001	0.01±0.005	nd
		STD	14	0.06±0.002	0.14±0.001	0.02±0.001
1-heptanol	11.103	MS+	0	0.03±0.001	0.03±0.002	0.02±0.001
		STD	14	0.02±0.001	0.01±0.001	0.03±0.001
1-octen-3-ol	11.349	MS+	0	0.08±0.03	0.03±0.01	0.01±0.001
		STD	14	0.01±0.001^b^	0.14±0.006^a^	0.14±0.001^a^
2-nonen-1-ol	13.372	MS+	0	0.03±0.001	0.01±0.001	0.01±0.001
		STD	14	0.02±0.001	0.02±0.001	0.01±0.001
1-octanol	13.452	MS+	0	nd	0.02±0.001^B^	0.02±0.002
		STD	14	nd	0.04±0.001^A^	0.03±0.001
1-hexanol	8.316	MS+	0	0.04±0.003	0.02±0.002	0.02±0.002
		STD	14	0.02±0.001	0.02±0.001	0.02±0.001
1-undecanol	17.431	MS	0	nd	0.01±0.001	0.03±0.001
			14	nd	0.01±0.001	0.01±0.001
Pyrazines						
Pyrazine, methyl-	6.92	MS+	0	nd	nd	0.03±0.001
		STD	14	nd	0.04±0.001	0.06±0.002
Pyrazine, 2,4-dimethyl-	9.678	MS+	0	nd	nd	0.01±0.002
		STD	14	nd	0.05±0.001	0.04±0.001
Pyrazine, 3-ethyl-2,5-dimethyl-	13.581	MS+	0	0.02±0.001	0.02±0.001^B^	0.04±0.001^B^
		STD	14	0.01±0.002^c^	0.06±0.002^Ab^	0.10±0.03^Aa^
Pyrazine,2,3-dimethyl-	9.529	MS	0	nd	nd	0.01±0.003
			14	nd	0.14±0.003	0.09±0.003
Trimethyl pyrazine	11.795	MS+	0	nd	0.04±0.001^b^	0.13±0.001^a^
		STD	14	nd	0.06±0.001^b^	0.15±0.001^a^
Sulfur-containing compounds						
Carbon disulfide	1.864	MS+	0	0.01±0.001	0.01±0.001	0.01±0.001
		STD	14	nd	nd	nd
2-methylthiophene	5.053	MS+	0	nd	0.07±0.001	0.06±0.12
		STD	14	nd	0.08±0.001	0.07±0.003
2-thiophene methanol	12.561	MS+	0	0.02±0.001	0.02±0.001	0.01±0.002^B^
		STD	14	0.03±0.001	0.04±0.001	0.04±0.002^A^
3-thiophene-carboxaldehyde	11.663	MS	0	nd	0.02±0.001	0.01±0.001
			14	nd	0.07±0.001	0.02±0.001
3-phenyl thiophene	19.657	MS+	0	0.04±0.001	0.02±0.001^A^	0.03±0.004
		STD	14	0.02±0.002	0.01±0.001^B^	0.02±0.001
Furans						
2-pentylfuran	11.584	MS+	0	0.12±0.05	0.01±0.001	0.01±0.001
		STD	14	0.04±0.001	nd	nd
2-n-octylfuran	17.814	MS+	0	0.05±0.002	0.09±0.003^A^	0.08±0.001^A^
		STD	14	0.04±0.001	0.04±0.001^B^	0.04±0.006^B^
Hydrocarbons 4-octanone	11.134	MS	0	0.07±0.003	0.01±0.001	nd
			14	0.03±0.002	nd	nd
Heptane, 3,4-dimethyl-	13.037	MS	0	0.02±0.001	0.01±0.001	0.01±0.002
			14	0.01±0.002^b^	0.02±0.001^b^	0.03±0.001^a^
Toluene	4.94	MS+	0	0.01±0.001	0.01±0.001	0.01±0.001
		STD	14	0.02±0.001	0.01±0.004	0.02±0.001
Z-3-dodecene	14.706	MS	0	0.08±0.001	0.05±0.001	0.06±0.001
			14	0.03±0.001	0.04±0.001	0.04±0.002
2,5-octanedione	11.408	MS+	0	0.07±0.02	nd	0.02±0.001
		STD	14	0.04±0.001	0.02±0.001	0.05±0.002
2-heptanone	8.894	MS+	0	0.01±0.001	0.03±0.002	0.01±0.001
		STD	14	0.02±0.001	0.04±0.001	0.02±0.001
1,2,3-trimethylbenzene	12.307	MS	0	0.05±0.001	nd	0.02±0.001
			14	0.01±0.001	0.01±0.002	0.01±0.001
2-Heptene, 5-methyl-	6.508	MS	0	0.05±0.001	0.02±0.001^B^	0.02±0.002
			14	0.03±0.002^b^	0.10±0.001^Aa^	0.01±0.001^b^
Tridecane	17.952	MS	0	0.06±0.001^Ab^	0.009±0.001^Ba^	0.02±0.001^b^
			14	0.01±0.001^B^	0.01±0.006^A^	0.01±0.001
2-tridecanone	21.265	MS	0	0.07±0.001^a^	0.03±0.001^Ab^	0.03±0.001^b^
			14	0.02±0.001	0.01±0.001^B^	0.02±0.001
Hexadecane	22.782	MS	0	0.04±0.001^a^	0.01±0.001^b^	0.02±0.001^b^
			14	0.01±0.002	0.01±0.001	0.01±0.001

nd, not detectable.

1) IM, identification method: The compounds were identified by either mass spectra (MS) from library or authentic standards (STD).

2) SV, sous vide cooking at 55°C for 5 h and raised to 60°C for 1 h; SV+OV, sous vide cooking followed by oven roasting at 250°C for 20 min; SV+TC, sous vide cooking followed by blowtorching for 2 min.

Means within a same row with different superscripts (^a–c^) differ significantly at (p<0.05).

Means within a same column in each parameter with different superscripts (^A,B^) differ significantly at (p<0.05).

**Table 5:** Total amount of flavor class in beef striploin steaks as affected by ageing and cooking treatment

Flavor class	Ageing (d)	SV1)	SV+OV1)	SV+TC1)
Aldehydes	0	4.24±0.06^b^	4.82±0.12^ab^	4.59±0.09^a^
	14	2.01±0.03^b^	3.56±0.08^a^	3.12±0.05^a^
Alcohols	0	0.36±0.01^a^	0.13±0.001^b^	0.12±0.001^b^
	14	0.13±0.001	0.40±0.003	0.28±0.02
Pyrazines	0	0.02±0.001^c^	0.06±0.001^b^	0.22±0.008^a^
	14	0.01±0.001^b^	0.35±0.01^ab^	0.44±0.05^a^
Sulfur-containing compounds	0	0.07±0.001^b^	0.14±0.002^a^	0.12±0.001^a^
	14	0.05±0.001^b^	0.2±0.0.6^a^	0.15±0.003^a^
Furans	0	0.17±0.01	0.1±0.005	0.09±0.001
	14	0.08±0.001	0.04±0.001	0.04±0.001
Hydrocarbons	0	0.53±0.01^a^	0.17±0.004^b^	0.22±0.002^b^
	14	0.29±0.002	0.32±0.002	0.27±0.0003

1) SV, sous vide cooking at 55°C for 5 h and raised to 60°C for 1 h; SV+OV, sous vide cooking followed by oven roasting at 250°C for 20 min; SV+TC, sous vide cooking followed by blowtorching for 2 min.

Means within a same row with different superscripts (^a–c^) differ significantly at (p<0.05).

Means within a same column in each parameter with different superscripts (^A,B^) differ significantly at (p<0.05).

**Table 6:** Sensorial mean score (7-point scale) of beef striploin steaks as affected by ageing and cooking treatment

Items	Ageing (d)	SV1)	SV+OV1)	SV+TC1)
Flavor	0	3.79±0.10^Bc*^	5.65±0.12^b^	6.25±0.11^Ba^
	14	4.63±0.14^Ac^	5.47±0.15^b^	6.50±0.11^Aa^
Juiciness	0	5.83±0.10^a^	4.94±0.10^Ab^	5.55±0.12^a^
	14	5.84±0.09^a^	5.53±0.13^Bb^	5.90±0.09^a^
Tenderness	0	5.63±0.14^aB^	5.24±0.11^b^	5.56±0.12^abB^
	14	5.97±0.16^aA^	5.18±0.12^b^	6.25±0.07^aA^
Overall acceptability	0	4.74±0.12^cB^	5.59±0.14^b^	6.35±0.11^Ba^
	14	5.12±0.11^bA^	5.12±0.13^b^	6.50±0.10^Aa^

1) SV, sous vide cooking at 55°C for 5 h and raised to 60°C for 1 h; SV+OV, sous vide cooking followed by oven roasting at 250°C for 20 min; SV+TC, sous vide cooking followed by blowtorching for 2 min.

* Means±standard errors; the mean values were calculated using 7-point scale (7 = extremely like; 6 = like very much; 5 = like moderately; 4 = neither like nor dislike; 3 = dislike moderately; 2 = dislike very much; and 1 = dislike extremely).

Means within a same row with different superscripts (^a–c^) differ significantly at (p<0.05).

Means within a same column in each parameter with different superscripts (^A,B^) differ significantly at (p<0.05).
